# High thermal plasticity, low heritability and constant directional selection on breeding phenology in a viviparous ectotherm

**DOI:** 10.1111/1365-2656.70335

**Published:** 2026-08-14

**Authors:** Théo Bodineau, Alexis Rutschmann, Jean‐François Le Galliard, Manuel Massot, Murielle Richard, Jean Clobert, Sandrine Meylan, Pierre de Villemereuil

**Affiliations:** ^1^ Institut d'Ecologie et des Sciences de l'Environnement de Paris (iEES Paris—UMR 7618), Sorbonne Université, UPEC, UPCité, CNRS, INRAE, IRD Paris France; ^2^ Station d'Ecologie Théorique et Expérimentale de Moulis, CNRS UAR2029 Moulis France; ^3^ École Normale Supérieure, Département de Biologie CNRS, UAR 3194, Centre de Recherche en Ecologie Expérimentale et Prédictive (CEREEP‐Ecotron IleDeFrance), PSL Research University Saint‐Pierre‐lès‐Nemours France; ^4^ Institut de Systématique, Évolution, Biodiversité (ISYEB), École Pratique des Hautes Études, PSL University, MNHN, CNRS, SU, UA Paris France; ^5^ Institut Universitaire de France (IUF) France

**Keywords:** adaptation, climate change, ectotherm, phenology, phenotypic plasticity, quantitative genetics, reproduction, selection

## Abstract

1. Shifting breeding phenology is a widespread response to global warming in ectothermic species such as reptiles, with considerable impacts on reproductive success and population dynamics. Thus, understanding causes and consequences of plasticity and microevolution in breeding phenology is important for determining vulnerability to climate change.
2. Here, we study environmental and individual determinants of breeding phenology, its heritability and the temporal variation of selection on parturition date using a 31‐year monitoring of a wild, viviparous population of common lizards (*Zootoca vivipara*).
3. Warmer daily maximum temperatures during the post‐winter activity advanced parturition dates and larger females gave birth earlier, but warmer temperatures also reduced size‐related phenological variability. Parturition dates were repeatable but weakly heritable, suggesting that inter‐individual consistency of parturition dates may be related to non‐heritable factors such as home range quality, nutritional condition or behaviour. In addition, we found evidence for a directional selection towards earlier parturition without fluctuation in time.
4. This study highlights the strong inter‐annual thermal plasticity coupled with a limited adaptive potential of breeding phenology in the common lizard. In future, warmer conditions, the interplay between such plasticity and a selection apparently independent from environmental fluctuations would thus essentially depend on the yet unknown costs of thermal plasticity.

## Graphical abstract

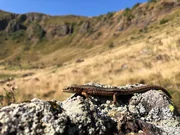

## 1 INTRODUCTION

For many organisms living in seasonal environments, breeding phenology is closely related to life history traits that determine population dynamics (e.g. annual growth, survival and reproductive success) and therefore to persistence in changing environments (Iler et al., 2021; McLean et al., 2016). Changes in breeding phenology related to warmer temperatures have been widely reported over the last decades and are considered as a widespread response to climate warming in natural populations of plants and animals (reviewed in Walther et al., 2002; Root et al., 2003; Parmesan, 2006). However, such warming‐induced phenological changes exhibit significant variation in both direction and magnitude across taxonomic groups (Thackeray et al., 2016; Vitasse et al., 2021), and their effects on individual fitness remain highly uncertain across taxa (Duputié et al., 2015). While stabilising selection on breeding phenology is documented in some birds and mammals (e.g. de Villemereuil et al., 2020), selection on breeding dates in other vertebrate taxa has received less attention so far (but see Olsson & Shine, 1997; Wapstra et al., 2010; Bogner et al., 2016). Yet, accurate quantification of the form and strength of selection on breeding phenology across a range of species is essential for reliably assessing the consequences of phenological shifts.

In squamate reptiles, and particularly in lizards, experimental and correlative studies on captive, semi‐natural or wild populations both show widespread evidence for directional selection favouring earlier laying or parturition dates, with many populations characterised by a seasonal decrease in reproductive investment and number of viable offspring (Bodineau et al., 2024; Mitchell et al., 2018; Olsson & Shine, 1997). This seasonal decline in fitness is similar to the one observed in other animal taxa (e.g. birds: Öberg et al., 2014; insects: Landa, 1992; mammals: Feder et al., 2008). One explanation, called the ‘Date hypothesis’, lies in the existence of a covariation between breeding dates and environmental conditions (e.g. food availability, mate quality, predation risk or temperature) thereby favouring early breeding in seasonal climates (Hatchwell, 1991; Moreno, 1998). Another non‐exclusive explanation is the ‘Quality hypothesis’, relying on a covariation between the quality of breeders and their breeding date (Brinkhof et al., 1993; Hatchwell, 1991). For example, both nutritional state and body condition are known to positively influence the timing and success of reproduction (Hadfield & Reed, 2022; Price et al., 1988; Verhulst & Nilsson, 2008). Yet, while some studies on birds and mammals have reported fluctuating selection on breeding date depending on climatic conditions or prey availability (de Villemereuil et al., 2020; Gamelon et al., 2018; Gienapp et al., 2013; Husby et al., 2011; Marrot et al., 2018; Visser et al., 1998, 2015), no study has quantified temporal changes in the strength of selection on breeding dates in reptiles so far. Quantifying both the form and fluctuation of natural selection could therefore improve our understanding of the selective pressures acting on phenology in highly temperature‐sensitive vertebrate species.

In temperate reptiles, inter‐annual variations in breeding phenology are mainly explained by the thermal plasticity of the reproductive cycle (e.g. Ljungström et al., 2015). Life history traits of ectotherms are particularly sensitive to external thermal conditions because of the tight relationship that exists between environmental temperatures, essential biochemical processes (e.g. biochemical reaction kinetics, enzyme inactivation or hormone regulation; see Hochachka & Somero, 2002) and behavioural activity patterns (Gunderson & Leal, 2015). That is, warmer temperatures during the reproductive cycle are known to advance spring emergence (Moss & MacLeod, 2022) as well as follicular and embryonic development (Duvall, 1982; Noble et al., 2018) and ultimately should thus advance laying or parturition dates (Ljungström et al., 2015; Rutschmann, Miles, Le Galliard, et al., 2016). One consequence of this profound influence of temperature on breeding traits is that reptiles' breeding phenology is likely to be sensitive to thermal conditions throughout the entire reproductive cycle (i.e. from post‐winter emergence to the end of gestation), rather than at a critical period of the reproductive cycle (e.g. Porlier et al., 2012). Environmental cues and the time‐window influencing breeding phenology have been well studied in birds (e.g. Andreasson et al., 2023; Drake & Martin, 2018), but climatic determinants of breeding dates remain poorly explored in reptiles. Due to their limited dispersal capacities, which constrain their ability to adjust phenology by shifting habitat, their breeding dates should be closely linked to the local climate and respond strongly to its variations.

In addition, studies in reptiles often report significant inter‐individual variations in reproductive timing (Cunningham et al., 2020; Ljungström et al., 2015; Olsson & Shine, 1997; Wapstra et al., 2010), but mechanisms underlying such inter‐individual variations are not well understood. In lizards, body size and condition can strongly influence reproductive phenology, with larger and higher‐condition females giving birth earlier (Bauwens & Verheyen, 1985; Hall et al., 2018; Le Galliard et al., 2010; Olsson & Shine, 1997). This pattern likely reflects the influence of body size, age or body condition on the energy available to fuel early reproduction, on the reproductive experience and on the competitive ability. However, the genetic basis of inter‐individual variation in reproductive phenology has been little investigated in reptiles compared to other vertebrates, hindering our understanding of the evolvability of this trait (reviewed in mammals: Boutin & Lane, 2014; birds: Charmantier & Gienapp, 2014; reptiles: Urban et al., 2014). Some studies on lizards report repeatable parturition dates among years (Cunningham et al., 2020; Wapstra et al., 2010), but quantifying the heritability of this critical reproductive trait remains crucial for determining the adaptive potential of populations facing climate change (Gulisija, 2026; Urban et al., 2024).

Here, we quantified climatic and individual determinants of parturition date, as well as the shape, strength and temporal variation of selection on parturition date using a long‐term mark‐recapture dataset (31 years) from a natural, viviparous population of European common lizards (*Zootoca vivipara*, Lichtenstein 1823) in France. First, in line with earlier studies showing that cold climatic conditions constrain activity and reproduction in this cool population in the southern margin of the species distribution (Chamaillé‐Jammes et al., 2006; Huey, 1982; Marquis et al., 2008), we expected mean daily maximum temperatures during the post‐winter activity period (including emergence, mating and early gestation) to strongly influence inter‐annual variations in parturition dates. Furthermore, we predicted larger females to give birth earlier than smaller ones, given the documented link between female body size and breeding phenology in lizards (e.g. Le Galliard et al., 2010; Olsson & Shine, 1997). Second, we expected low repeatability and heritability of absolute (i.e. calendar) parturition dates because inter‐annual variation in parturition date is strongly influenced by annual thermal conditions. In contrast, relative parturition dates, which reflect an individual's tendency to breed earlier or later than conspecifics, should exhibit higher repeatability (Cunningham et al., 2020; Wapstra et al., 2010) as well as higher heritability. Finally, we hypothesised a directional selection towards earlier parturition dates (Olsson & Shine, 1997), with an intensity that may fluctuate depending on the thermal conditions experienced by females during the reproductive cycle. Warmer environmental temperatures could indeed provide more favourable conditions for thermoregulation, embryonic development management and prey availability, thus reducing the strength of selection on parturition date.

## 2 MATERIALS AND METHODS

### 2.1 Model species, study site, sampling and rearing conditions

Longitudinal data on reproduction were obtained from long‐term mark‐recapture monitoring conducted from 1989 to 2019 in a population of common lizards located near the ‘Mas de la Barque’ (Mont‐Lozère, France; 1420 m a.s.l., see details in Clobert et al. (1994)). The European common lizard is a small lizard widely distributed in northern Eurasia and inhabiting highly seasonal, cool and humid habitats. In our study area, activity starts for adult males around mid‐April and for adult females in early May. In our study area, mating occurs immediately after female emergence and vitellogenesis occurs on average during the first 3 weeks of activity (Bauwens & Verheyen, 1985). Reproductive mode is viviparous and parturition occurs after an average gestation period of ca. 2 months (i.e. between July and early August (Bauwens & Verheyen, 1985)). Females lay on average five non‐calcified eggs (range from 1 to 12) including both nonviable offspring (fertilised or unfertilised eggs, undeveloped embryos and stillborn) and live ones which usually hatch within a few minutes to a few hours after parturition.

Each year, lizards were captured between early‐June and mid‐July ca. 1 month before the mean parturition date of females in this population (mean capture date: 29th June ±12.5 SD). Variations in capture dates among years are mainly due to variations in weather conditions, which influence capture possibilities and efficiency. Lizards were identified or marked by toe‐clipping, measured for body length (snout‐vent length, SVL) and sampled for tail tip tissues (<1 cm) for pedigree construction (see below). Immediately after being measured, all individuals were released at the capture location except gravid females which were transported to the laboratory nearby the field site and kept until parturition. We maintained females with ad libitum food and water in individual terrariums (18 × 11 × 12 cm). Each terrarium was filled with sterilised peat as substrate and equipped with a shelter. Terrariums were lit automatically between 9:00 AM to 12:00 PM and 2:00 PM to 5:00 PM by an incandescent heating bulb (25 W) placed on one side of the terrarium to generate a thermal gradient. After parturition, the litter was characterised by recording the number of unfertilised eggs, undeveloped embryos, stillborn and live juveniles. Live juveniles were marked by toe‐clipping and a tail tip tissue was collected (<3 mm) to infer paternity (see below). All juveniles were released within 3 days after birth, together with their mothers, at the mother's capture location. Note that the rearing site changed four times during the long‐term monitoring due to logistical constraints (respectively, in 1993, 1997 and 2002). Differences in rearing conditions were taken into account in the statistical analyses (see below) to correct for any temperature variation resulting from differences in building insulation and location, which could affect parturition date. Experiments were performed under the approval of Cuvier Experimentation Ethics Committee (APAFIS#15897‐2018070615164391 v3 & APAFIS #34093‐2021112211094302 v4) in an animal breeding facility accredited by the Departmental Directorate for Employment, Labour, Solidarities and Population Protection with permit DDETSPP‐SPAE‐2022‐262‐001.

### 2.2 Climate data collection

Local climatic conditions (1989–2019) were reconstructed using weather conditions recorded by the nearest, high‐quality, long‐term meteorological station, which is situated at a similar elevation but 50 km south of our study population (Météo France, Mont Aigoual, ID: 30339001, 1.567 m a.s.l., 44°07′17″ N, 3°34′53″ E). To do so, data collected at Mont Aigoual between August 2022 and 2023 were downscaled to local conditions using climatic data collected in situ with HOBO® Pro v2 U23‐002 loggers (ONSET 188 Cape Cod, Massachusetts) installed over the same time period in the shade, within a solar radiation shield. Specifically, we used linear regressions to relate the mean daily minimum, mean and maximum temperatures recorded in our study site and the ones recorded at the Mont Aigoual from August 2022 to 2023 (*n* = 356; *R* ^2^ = 0.82, *R* ^2^ = 0.94 and *R* ^2^ = 0.96, respectively; Figure S2). We then used the regression coefficients from each linear regression to reconstruct historic mean daily minimum, mean and maximum temperatures in our study site from Mont Aigoual data recorded over the entire time series (1989–2019). This method is based on the assumption of a stable relationship between Mount Aigoual and local temperatures over time, but provides a reliable reconstruction of the local climate at our study site (Le Galliard et al., 2010).

### 2.3 Pedigree

The genetic pedigree of the population was reconstructed from DNA samples extracted from caudal tissue samples from all captured animals and juveniles using DNeasy Blood & Tissue (Qiagen) kit. We genotyped microsatellite markers at 8 loci (Richard et al., 2012). The CERVUS software (Kalinowski et al., 2007) was then used to infer paternity of each newborn based on probabilistic modelling of allele segregation. When the individual was not born in the laboratory, we assigned fictitious identities to unknown (i.e. uncaught) parents. As the depth of the pedigree was unsatisfactory before 2007, we carried out heritability analyses on a subset dataset between 2007 and 2019. Over this period, the pedigree had a maximum depth of 5 with an average of 1.60 generations.

### 2.4 Statistical analysis

Analyses were performed using R software version 4.2.0 (R Core Team, 2022) on a dataset containing 2119 female breeding events between 1989 and 2019, except for the estimation of heritability coefficients that were performed on a subset of the dataset with 691 female breeding events (see Table 1). In all cases, we tested model fitting, model assumptions and we compared models using the Akaike information criterion corrected for small sample size (AICc).

**TABLE 1:** Variance decomposition, repeatability and heritability estimates of female relative parturition date in a natural population of common lizards (*Zootoca vivipara*).

	Parameter	Posterior mean	Posterior median	95% credible interval
Repeatability	Individual identity variance	0.079	0.079	0.054–0.108
	Fixed effects variance	0.026	0.025	0.014–0.041
	Year variance	0.728	0.676	0.375–1.38
	Residual variance	0.154	0.153	0.133–0.178
	Total phenotypic variance	0.987	0.936	0.632–1.64
	**Repeatability of relative parturition date**	**0.305**	**0.305**	**0.220–0.389**
Heritability	Additive genetic variance	0.037	0.037	0.000220–0.085
	Fixed effects variance	0.030	0.029	0.018–0.0439
	Individual identity variance (permanent environment variance)	0.036	0.034	0.000252–0.0856
	Mother identity (maternal‐effect variance)	0.017	0.012	0.0000336–0.0573
	Year variance	0.783	0.689	0.330–1.81
	Residual variance	0.131	0.132	0.0952–0.165
	Total phenotypic variance	1.03	0.94	0.579–2.06
	**Heritability of relative parturition date**	**0.147**	**0.148**	**0.000898–0.330**

*Note*: To estimate the repeatability and heritability of the relative parturition date, we subtracted the year variance from the total phenotypic variance. The repeatability of parturition dates has been estimated on the whole dataset from 1989 to 2019 (2119 breeding events) and the heritability has been estimated on a subset of the dataset from 2007 to 2019 (691 breeding events). The relative repeatability of parturition dates, estimated on a subset of the dataset from 2007 to 2019, closely matched full‐dataset estimates (posterior mean: 0.313; posterior median: 0.313; 95% credible interval: 0.191–0.433). Bold values indicate estimates of repeatability and heritability.

#### 2.4.1 Climatic and individual determinants of parturition dates

We estimated phenological trends in mean parturition dates (expressed as day of the year) from 1989 to 2019 with a linear model fitted with rearing site (to control for rearing site changes) and time (years) as fixed effects. We tested both linear and quadratic effects of time to assess the best fit for temporal trends. Then, we carried out a sliding window analysis to determine whether there was a period during which this species was most strongly influenced by thermal conditions. We focused on the influence of environmental temperatures on parturition dates by generating 4, 6, 8, 10 or 12‐weeks time‐windows at sliding 2‐week intervals. These periods were generated between 1st April (i.e. before likely post‐winter emergence) and 12th July (i.e. the average date of the end of the capture period). For each time‐window, we compared the effects of mean daily minimum, mean and maximum temperatures on parturition dates using linear mixed models based on the *lme4* package (Bates et al., 2015). Models included capture date (assuming that females caught earlier give birth earlier due to warmer and more stable conditions in the rearing facility), rearing site and female body size as fixed effects. Individual identity and year were also included as random effects. To account for multiple testing in the sliding window analysis, we implemented a permutation procedure following (Macphie et al., 2026). Within each permutation, yearly climate data (i.e. all climate windows calculated for a given year) were randomly reassigned among years, such that all individuals from a given year received the same (but randomly assigned) climate values. This approach preserves within‐year structure and dependencies while removing the biological link between climate and parturition dates. For each permutation (*n* = 1000), the sliding window analysis was repeated and the minimum ΔAIC recorded to generate a distribution of minimum ΔAICs expected in the absence of any real effect of climate on parturition dates. The observed minimum ΔAIC (from the real data set) was then compared to this distribution using an empirical cumulative distribution function (*ecdf* function in R) to estimate the 2.5% and 97.5% quantiles and to calculate the probability of obtaining the observed minimum ΔAIC under the null hypothesis. This model selection led to retain mean daily maximum temperatures between 29th April and 7th July (hereinafter called *T*_max_) as the best explanatory temperature proxy (see below). Since phenological advances caused by higher temperatures can be accompanied by a decrease in parturition dates variance (Massot et al., 2017), we also characterised changes in parturition date variances as a function of *T*_max_ by adding a power variance function (‘varPower’ in *nlme* package) to the best model previously selected by the sliding window analysis. Finally, we also tested whether size‐induced phenological differences were influenced by *T*_max_ by incorporating an interactive effect of female body size and *T*_max_.

#### 2.4.2 Repeatability and heritability of parturition dates

We used the Bayesian R package *brms* with default priors (Bürkner, 2017) to estimate the repeatability and the heritability of parturition date. Number of iterations and length of burn‐in period were determined so that the effective sample size is above 1000 for all parameters and the potential scale reduction factor is 1.01 or below, after graphically checking for convergence (Vehtari et al., 2021). In general, studies in birds and mammals quantify the repeatability and heritability of the absolute (i.e. calendar) laying or parturition date, as the reproductive phenology of these species is first‐order constrained by photoperiod (Helm et al., 2013; Paul et al., 2007). However, because breeding phenology of reptiles is primarily determined by thermal conditions, which can fluctuate considerably each year (Van Dyke, 2014), we also calculated the repeatability and heritability of relative parturition date (i.e. the propensity of a female to give birth early or late relative to other females in the population). To quantify repeatability, we fitted a linear mixed model including body size and capture date as fixed effects and individual identity and the year as random effects. Models were run for 3000 iterations without thinning, after a burn‐in of 1000. We calculated the absolute parturition date repeatability as the ratio between the individual identity variance and the total phenotypic variance (i.e. sum of all variance components in the model). We calculated the relative parturition date repeatability as the ratio between the individual identity variance and the total phenotypic variance minus the between‐year variance.

We further estimated the heritability of parturition date using animal models that use the information on individuals' relatedness computed from the population pedigree (Kruuk, 2004; Thompson, 1976). We analysed parturition date as a Gaussian trait and included body size and capture date as fixed effects. In addition to the additive genetic effect, the female identity (permanent environment effect), the mother identity (maternal effect) and the year were fitted as random effects. The models were run for 10,000 iterations with a thinning interval of 5, after a burn‐in of 1000. The heritability of absolute parturition date was calculated as the ratio between the additive genetic variance and the total phenotypic variance. The heritability of the relative parturition date was calculated as the ratio between the additive genetic variance and the total phenotypic variance minus the between‐year variance, respectively (de Villemereuil et al., 2019). All derived estimates (e.g. repeatability or heritability) posterior distribution were computed using the full MCMC sample, thus accounting for the uncertainty in estimating variances in the model.

#### 2.4.3 Form and strength of selection on parturition dates

In order to quantify the shape and strength of selection on reproductive phenology, we used the number of viable juveniles produced by the female as an annual measure of female fitness. First, we investigated the shape of selection on parturition using zero‐inflated Poisson mixed models with the *glmmTMB* package (Brooks et al., 2017). We compared a null model (‘No selection’) to models including linear (‘Constant directional selection’) or quadratic effects of standardised parturition date (‘Constant non‐linear selection’) on the entire time series. Standardised parturition dates were calculated as the residuals of a multiple linear regression of the parturition date, from capture date and rearing site standardised (mean‐centred and standardised to a variance of 1) over the entire time series to correct for the effects of capture date and rearing site on parturition dates. In all models tested, we included the female body size which strongly constrains litter size in this species (Pilorge et al., 1983) and the rearing site as fixed effects. We also included a random effect of the year. Due to the relative scarcity of repeated measurements and based on a likelihood ratio test (LRT), the individual identity was not included in the random part of the model. Once the best model was chosen (i.e. ‘Constant directional selection’, see results), we investigated the temporal fluctuation of directional selection gradients between years by adding to the previous model an interaction term between the standardised parturition date and the year in the random part of the model (‘Fluctuating directional selection’). Third, since our analyses suggest the existence of constant directional selection (see Section 3), we attempted to discriminate the relative contribution of parturition date and climate on fitness. To do so, we compared the ‘Constant directional selection’ model to an alternative model (‘Constant selection mediated by *T*_max_’) in which we replaced the standardised parturition date with *T*_max_ identified as the main thermal driver of parturition dates and a model (‘Constant selection mediated by parturition date and *T*_max_’) including the combined effects of the standardised parturition date and *T*_max_. Statistical results of the natural selection analysis are reported in Table 2 and Table S1.

**TABLE 2:** Natural selection on breeding phenology.

Hypothesis	Model parameterization	*k*	AICc	ΔAICc	*w*_*i*_	LL
No selection	Annual number of viable juveniles ~ SVL + rearing site + (1|Year)	7	8774.0	5.85	0.02	−4380.0
**Constant directional selection**	**Annual number of viable juveniles ~ SVL + standardised parturition date + rearing site + (1|Year)**	**8**	**8768.2**	**0**	**0.43**	**−4376.0**
Constant quadratic selection	Annual number of viable juveniles ~ SVL + standardised parturition date + standardised parturition date^2^ + rearing site + (1|Year)	9	8770.0	1.84	0.16	−4375.9
Fluctuating directional selection	Annual number of viable juveniles ~ SVL + standardised parturition date + rearing site + (standardised parturition date|Year)	10	8769.6	1.45	0.21	−4376.0
Constant selection mediated by *T*_max_	Annual number of viable juveniles ~ SVL + *T*_max_ + rearing site + (1|Year)	8	8775.6	7.47	0.01	−4379.7
Constant selection mediated by parturition date and *T*_max_	Annual number of viable juveniles ~ SVL + standardised parturition date + *T*_max_ + rearing site + (1|Year)	9	8770.1	1.91	0.16	−4375.9

*Note*: AICc‐based model selection comparing the influence of parturition date and mean daily maximum temperatures from 29th April to 7th July (*T*_max_) on the annual number of viable juveniles (i.e. annual reproductive success) produced by female common lizard (*Zootoca vivipara*). Body size and rearing site were always included in the model, which also accounts for random variation in the intercept (mean value) among years. Random effects are indicated in brackets. Bold values indicate the selected best model.

Abbreviations: AICc, Akaike information criterion; *k*, number of parameters; LL, log likelihood; SVL, snout‐vent length; *T*_max_, mean daily maximum temperature between 29th April and 7th July; *w*_*i*_, AICc weight; ΔAICc, AIC difference between ranked models.

## 3 RESULTS

### 3.1 Climatic and individual determinants of parturition dates

The range of average parturition date was between the 10th July and the 10th August over the period 1989–2019. Mean daily maximum temperatures between the 29th April and 7th July of the breeding year (*T*_max_) were the temperatures best explained the inter‐annual variation in parturition dates over the past 31 years (proportion of annual variance explained = 74%; AICc weight: 0.39; see full list of AICc in available data). The observed minimum ΔAIC for this climate window (ΔAIC = −36.3) fell outside the 95% confidence interval of the permuted minimum ΔAIC distribution ([CI: −9.77 to 1.28]), yielding a probability of obtaining the observed minimum ΔAIC under the null hypothesis equal to 0. On average, higher *T*_max_ resulted in earlier parturition dates with approximately a 4‐day advancement per degree increase (*b* = −3.97 ± 0.50 SE; *χ* ^2^_1_ = 62.9, *p* < 0.0001; Figure 1). This suggests that the inter‐annual variability of parturition dates in this population is mainly explained by *T*_max_ during the post‐winter activity period encompassing both vitellogenesis (in May) and mid‐gestation (in June–July) rather than by a more restricted time‐window. Additionally, model fitting improved when accounting for heterogeneous variance across *T*_max_, with evidence that the variance of parturition dates decreased with warmer *T*_max_ (LRT = 47.8, df = 1, *p* < 0.0001). Furthermore, parturition dates were significantly influenced by the interactive effect of *T*_max_ and female body size (*b* = 0.24 ± 0.08 SE; *χ* ^2^_1_ = 9.6, *p* = 0.002; Figure 2). While larger females gave birth earlier than smaller females on average, the correlation with body size was attenuated as the *T*_max_ increased. From 1989 to 2019, *T*_max_ increased by around 2.5°C in our study site (*b* = 0.08 ± 0.03 SE; *F*_1,29_ = 5.46, *p* = 0.005; Figure S1a), but we found no significant trend for mean parturition dates (linear change: *F*_1,26_ = 0.03, *p* = 0.87; quadratic change: *F*_1,25_ = 0.26, *p* = 0.61; Figure S1b).

**FIGURE 1:**
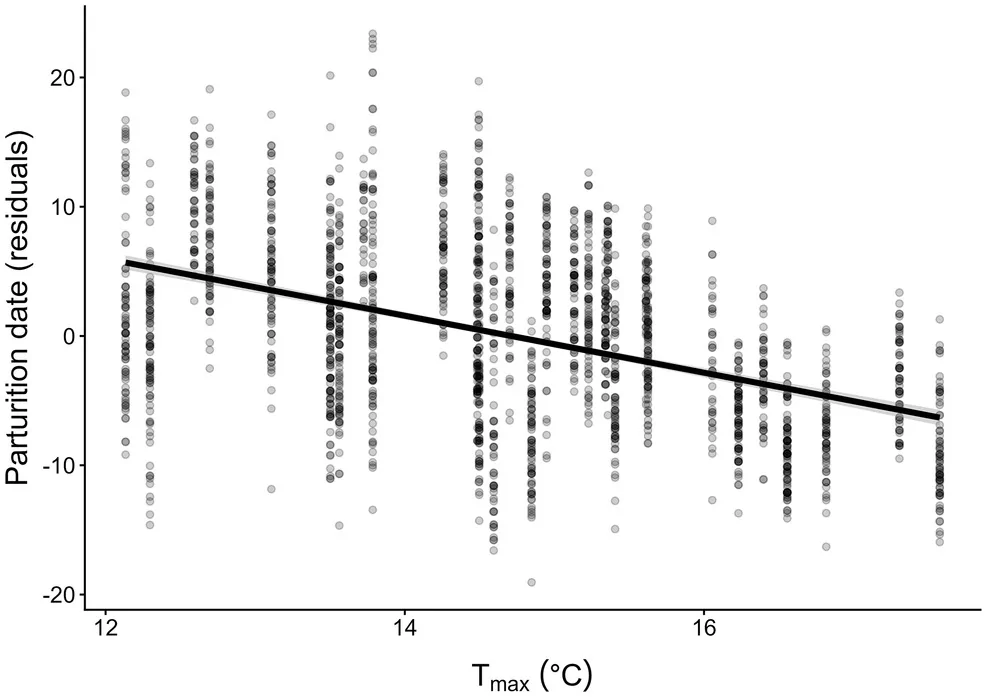
Relationship between female parturition date (residuals of a linear regression between female parturition date, capture date and rearing site) and mean daily maximum temperatures from the 29th April to the 7th July (*T*_max_). Points represent individual data and regression lines are displayed with their 95% confidence bands.

**FIGURE 2:**
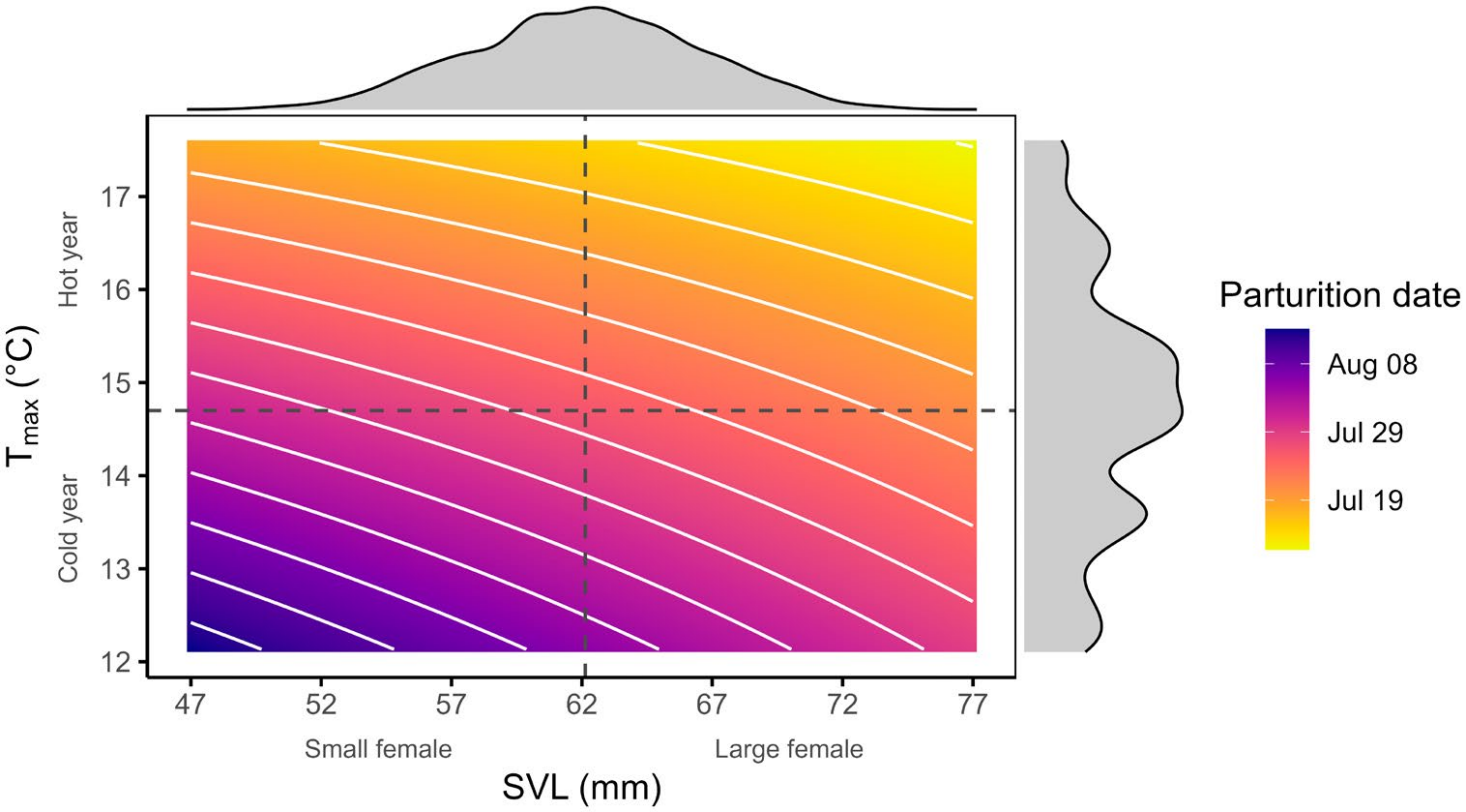
Parturition date as a function of female snout‐vent‐length (SVL) and mean daily maximum temperatures from the 29th April to the 7th July (*T*_max_). The surface represents the interpolation of parturition date values for each female SVL and mean daily maximum temperature calculated from the best‐selected model. The colour gradient highlights the values of predicted parturition date from late (blue) to earlier (yellow) parturition date. White contours show isoclines for parturition date. Dashed grey lines represent average conditions for mean daily maximum temperatures and female body size. Marginal density plots illustrate the observed distributions of female SVL and *T*_max_. Size‐induced phenological differences are more pronounced in colder years with lower daily maximum temperatures.

### 3.2 Variance decomposition, repeatability and heritability of parturition dates

Parturition dates were highly variable among years, with year variance accounting for 76% [CI: 57–88] of the trait variance. Relative parturition dates of individual females were repeatable (*r* = 0.31 [CI: 0.22–0.39]; Table 1), suggesting that females differed consistently from one another in their breeding phenology. Heritability was estimated on a subset dataset (i.e. 2007–2019) due to pedigree limitations. The heritability of the relative parturition date accounted approximately for roughly half of its repeatability, although showing large credible intervals (*h* ^2^ = 15% [CI: 0.09–33]; Table 1). In addition, repeatability and heritability values of absolute parturition dates (i.e. calendar parturition dates) were very low (*r* = 9% [4–14]; *h* ^2^ = 4% [0.02–10.8]). The estimated variance of the permanent environment effect on relative parturition date was close to the additive genetic variance and accounted for 14% [CI: 0.22–20] of the total phenotypic variance. The maternal‐effect variance explained a small amount (7% [CI: 0.02–13]) of the total phenotypic variance.

### 3.3 Form and strength of selection on parturition dates

We found a constant negative directional selection favouring an earlier parturition date (Table 2). Females who gave birth earlier produced more viable juveniles during the same year (*β* = −0.05 ± 0.02 SE; *χ* ^2^_1_ = 7.62, *p* = 0.006; Figure 3). Additionally, the number of viable juveniles at birth was positively correlated with female body size (*b* = 0.25 ± 0.01 SE; *χ* ^2^_1_ = 356.7, *p* < 0.0001). Furthermore, when comparing the main or combined effects of parturition date and *T*_max_ on the number of viable juveniles produced, we found that the best model explaining inter‐individual variations in fitness includes parturition date rather than the *T*_max_ or combined effects of both (Models ‘Constant selection mediated by *T*_max_’ and ‘Constant selection mediated by parturition date and *T*_max_’; Table 2), suggesting that parturition date has a more direct impact on the reproductive component of fitness than *T*_max_ per se.

**FIGURE 3:**
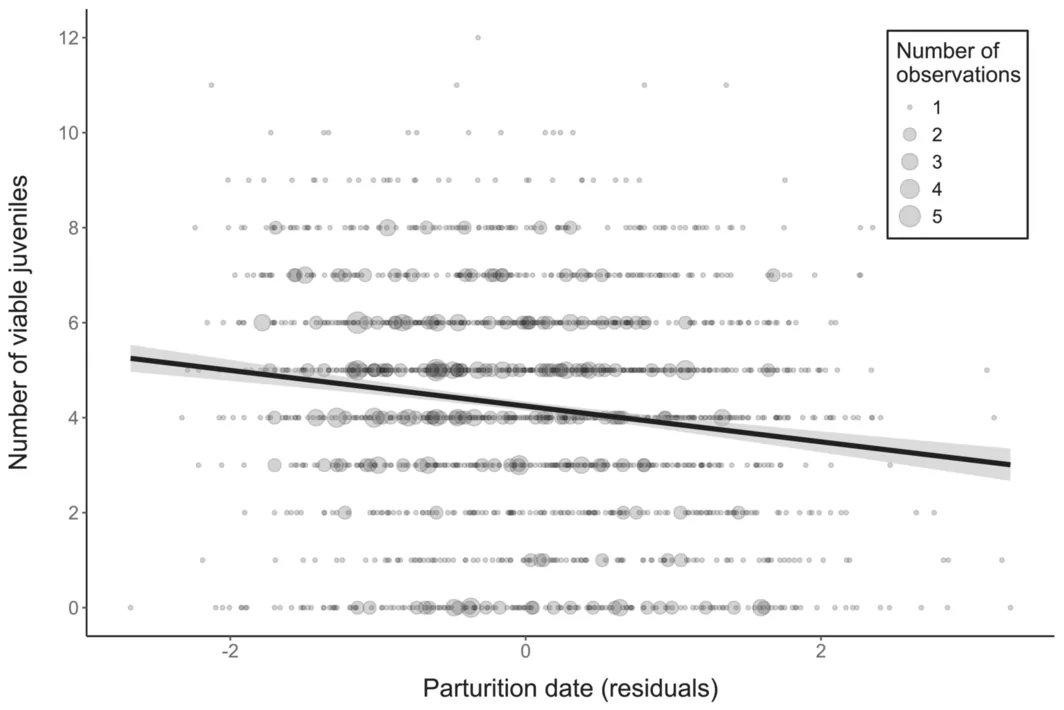
Standardised linear selection gradient for parturition date (solid lines) estimated from 31‐year long‐term monitoring of a natural population of common lizard (*Zootoca vivipara*). The parturition date corresponds to the standardised residuals of a multiple linear regression between the parturition date, capture date and rearing site. The linear regression is plotted with its 95% confidence limits (shaded areas) and the point size corresponds to the number of observations.

## 4 DISCUSSION

In this study we quantified individual and environmental determinants of breeding phenology and analysed patterns of natural selection acting on parturition dates over a 31‐year period in a wild population of a viviparous ectotherm. Our analyses revealed that inter‐annual variations in parturition dates were strongly associated with mean daily maximum temperatures during the female vitellogenesis and gestation period. Higher mean daily maximum temperatures were correlated with earlier parturition dates as well as a reduced inter‐individual variability of parturition dates. While we also found that larger females gave birth earlier on average than smaller ones, the strength of this relationship decreased as mean daily maximum temperatures increased. In addition, there was a constant, positive directional selection towards earlier parturition dates regardless of the environmental conditions encountered during reproduction. Finally, parturition dates were repeatable and weakly heritable, suggesting a low capacity of the breeding phenology to respond to natural selection.

### 4.1 Directional selection drives parturition date in the common lizard

Reproductive phenology is a critical trait influencing annual fitness in many mammal and bird species (reviewed in de Villemereuil et al., 2020). Although the number of viable juveniles used as fitness proxy represents only one component of it, the seasonal decline in the number of viable juveniles produced by females suggests a directional selection favouring earlier parturition dates, in line with previous studies in lizards (Bodineau et al., 2024; Mitchell et al., 2018; Olsson & Shine, 1997). Further, although developmental thermal conditions can affect the quality of embryonic development and the viability of juveniles produced (Noble et al., 2018; While et al., 2018), inter‐annual variations in annual reproductive success were correlated with the mean parturition date rather than with post‐winter daily maximum temperatures. Two non‐exclusive hypotheses have been formulated to explain the seasonal decline of fitness: the ‘*date hypothesis*’ (Hatchwell, 1991; Moreno, 1998) and the ‘*quality hypothesis*’ (Brinkhof et al., 1993; Hatchwell, 1991). The ‘*date hypothesis*’ predicts an environment‐dependent selection occurring through seasonal changes in environmental conditions such as climatic conditions and abundance of prey, sexual partners, predators or breeding territories (Hatchwell, 1991; Moreno, 1998). Instead, the ‘*quality hypothesis*’ predicts a seasonal decline in reproductive performance through differences in parental quality between early and late breeders. Higher‐quality individuals—for example, because they are better able to acquire food, secure high‐quality territories or obtain superior mates—breed earlier and outcompete lower‐quality individuals through inter‐individual competition (discussed in Verhulst & Nilsson, 2008; Harriman et al., 2017; Pärt et al., 2017). The respective contributions of environment‐ and quality‐dependent selection are generally difficult to disentangle without experimental manipulation of phenology (see Bodineau et al., 2024; Verhulst & Nilsson, 2008) and may both contribute to an apparent seasonal decline in reproductive success. However, we should expect fluctuating selection across years: either because year‐to‐year environmental variations shift the timing of resource availability (under the date hypothesis) or because the onset timing of the best individuals or the intensity of inter‐individual competition fluctuates (under the quality hypothesis). Indeed, inter‐annual variations in environmental temperatures, food availability and competition should influence their reproductive investment, the embryonic development and consequently, the number of viable juveniles produced (Rutschmann, Miles, Clobert, & Richard, 2016; Wang et al., 2017). For example, numerous studies in birds have demonstrated that selection pressures on breeding phenology are altered by yearly environmental conditions such as annual temperature or prey availability (Chevin et al., 2015; de Villemereuil et al., 2020; Gienapp et al., 2014; Husby et al., 2011; Jantzen & Visser, 2023; Le Vaillant et al., 2021; Marrot et al., 2018; Visser et al., 2015). Here, despite substantial inter‐annual variations in both environmental conditions and mean parturition dates, the strength of directional selection remained remarkably stable between 1989 and 2019. This lack of fluctuating selection could suggest that the environmental and individual factors underlying natural selection are relatively constant from year to year or, more likely, that they combine to produce a stable pattern across years in the common lizard. In the second case, the two mechanisms would operate together. On one hand, year‐to‐year environmental variations would drive the overall annual shifts in phenology, which would determine the absolute timing and success of the earliest breeders. On the other hand, the constant shape of the seasonal decline would be better explained by the quality hypothesis, where a consistent sorting of individual quality and competitive ability would maintain a stable selection slope across years. Therefore, these two hypotheses might not be exclusive mechanisms but rather complementary components that jointly shape selection on breeding phenology.

### 4.2 Influence of thermal environment and body size on breeding phenology

In line with several studies on lizards, we highlighted a significant plasticity in breeding phenology, characterised by large inter‐annual variations in parturition dates (Olsson et al., 2018; Wapstra et al., 2010). Inter‐annual variation in parturition dates is largely due to thermal conditions during the post‐winter activity period, from emergence to gestation, as temperature significantly affects all stages of the reproductive cycle (Duvall, 1982; Lutterschmidt, 2012). Maximum daily temperatures from the 29th April to the 7th July were the best thermal index to explain inter‐annual variations in breeding, and warmer maximum daily temperatures advanced parturition dates, which is in agreement with previous studies in the common lizard (Le Galliard et al., 2010; Massot et al., 2017; Rutschmann, Miles, Le Galliard, et al., 2016). In general, daily maximum temperatures are correlated with insolation and are a better predictor of thermoregulatory opportunities than mean or minimum temperatures in populations where cold conditions limit behavioural activity (Huey, 1982; Marquis et al., 2008). Although mean daily maximum temperatures from the 29th April to the 7th July increased by +0.8°C per decade at our study site between 1989 and 2019, we did not find an advance in mean parturition dates over this period. Massot et al. (2017) estimated an average advance in parturition date of ca. 8.1 days between 1989 and 2001 in several natural populations of common lizards including our study site, but our results suggest that estimates of phenological trends are probably overestimated. The reasons for such differing phenological trends are two‐fold. First, reliable estimation of phenological trends requires long‐term studies, as extreme climatic events can significantly affect ectotherm phenology (Li et al., 2025; see Figure S1) and bias short‐term phenological trends analyses. Second, our ability to detect phenological trends may be limited by changes in breeding sites that occurred during the course of population monitoring (in 1993, 1997 and 2002; see Figure S1). More specifically, four distinct rearing sites were used successively due to logistical constraints, each potentially exhibiting slightly different thermal conditions due to variations in building insulation and location. These differences introduced additional variability in parturition dates, potentially obscuring long‐term phenological trends. Overall, these challenges highlight the inherent difficulty of studying phenological trends in reptiles, for which minor interventions—such as capture timing or thermal conditions during rearing—can significantly influence breeding phenology, compared to model species where phenological monitoring can be conducted in situ (e.g. tracking bird phenology *in natura* using artificial nest boxes during the nest‐building and incubation stages (Both et al., 2009)).

Moreover, as observed in many lizards species (Bauwens & Verheyen, 1985; Hall & Warner, 2017; Le Galliard et al., 2010; Rutschmann, Miles, Le Galliard, et al., 2016), we found larger females to give birth earlier than smaller ones. More interestingly, our models showed climate to interact with female body size to drive parturition dates, with size‐induced phenological differences being more pronounced in colder post‐emergence periods with lower daily maximum temperatures. Three non‐exclusive hypotheses may explain the interactive effects of body size and mean daily maximum temperatures on parturition date. Firstly, body size can influence heat regulation capacity of females and consequently the parturition date. In general, larger individuals heat up and cool more slowly than smaller ones due to their stronger thermal inertia and several studies on lizards have highlighted that larger body size has a positive effect on body heat retention (Gabirot et al., 2013; Gvoždík, 2002; Zamora‐Camacho et al., 2014). In highland populations where thermoregulation opportunities are limited, larger females with greater thermal inertia could maintain a body temperature that optimises embryonic development for longer periods, enabling them to give birth earlier. The advantage of greater thermal inertia in larger females would be reduced in warmer years (i.e. years offering more thermoregulation opportunities), which could explain the reduction of size‐induced parturition dates differences during warmer years. Secondly, size‐induced differences in parturition dates may be linked to the fact that larger and older females are more competitive. For example, they could initiate their reproductive cycle earlier due to their breeding experience (Rickwood et al., 2025), body reserves or sexual selection processes (e.g. male preference for larger females during mating (Bauwens & Verheyen, 1985)), they could be more efficient in capturing prey or in mobilising energy during vitellogenesis and gestation (e.g. egg formation during vitellogenesis (Price, 2017)), and they could have privileged access to optimal thermoregulation sites (Rusch & Angilletta Jr, 2017). Warmer daily maximum temperatures could reduce some of these competitive advantages of larger females, if warmer temperatures increased invertebrate abundance (Robinet & Roques, 2010) or the abundance of high‐quality basking sites. Thirdly, delayed breeding dates in smaller females may be the result of trade‐offs between resource allocation to fuel reproduction and body growth, as seen in a Belgium population of the common lizard (Bauwens & Verheyen, 1985). Since warmer environmental temperatures can increase insect density (Haridas et al., 2016), increase lizard activity periods (Adolph & Porter, 1993) and the efficiency of prey acquisition and assimilation (Van Damme et al., 1991), warmer years may reduce this allocation trade‐off between early growth and reproduction. This could enable smaller females to initiate reproduction earlier, thereby reducing size‐induced phenological differences among females (Rutschmann, Miles, Clobert, & Richard, 2016).

### 4.3 Repeatability and heritability of parturition dates

We quantified the repeatability of parturition dates and provided a measure of the heritability of this trait in a wild population of a reptile species. Relative parturition dates (i.e. the propensity of a female to give birth earlier or later than other females in the population) were repeatable (*r* = 31%) and weakly heritable (*h* ^2^ = 15%) in our study population. Heritability estimate for relative parturition date in the common lizard are comparable in magnitude to the heritability of laying or parturition date generally observed in birds and mammals (*h* ^2^ = 0.13–0.19; Clements et al., 2011; Charmantier & Gienapp, 2014) and may even be slightly lower, as these studies assess the heritability of absolute breeding dates. As expected, given the high thermal plasticity of parturition dates, the repeatability and heritability values of absolute parturition dates (i.e. calendar parturition dates) were very low (*r* = 9% [4–14]; *h* ^2^ = 4% [0.02–10.8]). Regardless of the method used to estimate the heritability of parturition dates, approximately half of the inter‐individual consistency of parturition dates was related to additive genetic effects, and half was related to non‐heritable factors. The non‐heritable part of the individual‐level effects of parturition dates could be linked to female physiological or behavioural phenotype and/or female home range quality which could affect their parturition date. For example, the condition‐dependent model of laying date in migratory birds predicts that females optimise their breeding phenology as a function of arrival body condition, arrival date and the ability to gain in condition in breeding areas (Descamps et al., 2011; Hennin et al., 2016; Rowe et al., 1994). Common lizards have a limited annual activity window and use resources stored or recently acquired during reproduction to fuel reproduction (Bleu et al., 2013). Thus, females in better condition or those able to rapidly improve their condition prior to reproduction may reproduce earlier. Earlier breeding also provides a longer period to recover energy before winter and could allow earlier breeding the following year (Bodineau et al., 2024). Furthermore, thermal physiology (e.g. thermal preference) and the quality of female home range (e.g. microclimate conditions, availability of thermoregulation sites, food availability) could influence key physiological processes in reproductive timing (e.g. follicular recruitment, embryonic development). Thus, thermal traits and the microhabitat may explain a significant part of the individual consistency of parturition date (Shutt et al., 2022; Struelens et al., 2018).

In addition, the low heritability of parturition dates found in this study suggests slow and limited adaptive potential in reproductive phenology. While limited heritability could, in theory, contribute to the absence of a significant temporal trend in parturition dates, the very high among‐year variation in parturition date suggests that phenotypic plasticity is likely to dwarf any evolutionary response to selection. Thus, our study supports the idea that plasticity plays a central role in reptile populations' responses to climate change. Plastic adjustments in reproductive phenology in response to rising temperatures could provide immediate fitness benefits—such as earlier parturition dates, which can increase the number of viable juveniles produced (this study) and confer further fitness advantages for juveniles with increased survival, growth and recruitment (Le Henanff et al., 2013; Li et al., 2018; Liu et al., 2022; Olsson & Shine, 1997; Qualls & Andrews, 1999; Uller & Olsson, 2010; Wapstra et al., 2010). Moreover, such plasticity could buy time for the emergence of evolutionary responses (Fox et al., 2019; Ghalambor et al., 2007). Nevertheless, a detailed quantification of the costs and benefits of this thermal plasticity in ectotherms remains largely unexplored. Few recent studies on reptiles have shown that thermal acceleration of gestation beyond optimal environmental temperatures can disrupt homeostasis in breeding females given the increased energetic costs of reproduction at high temperatures (e.g. reserve depletion (Bodineau et al., 2024; Brusch IV et al., 2023)). Therefore, while thermal plasticity may offer short‐term advantages, its long‐term benefits will depend on its costs, limitations and alignment with the direction of environmental change (Chevin et al., 2010). Overall, the low heritability of breeding dates, combined with high thermal plasticity, the potential costs of plastic responses and the inherent noise in our study design (discussed above), may explain the absence of an evolutionary response to selection observed in this population over the 31‐year study period.

## 5 CONCLUSION

Our findings reveal that breeding phenology of the common lizard is shaped by strong thermal plasticity, body size effects, constant selection for earlier parturition, but low heritability. This suggests that phenological responses to climate change in this species, and likely in other squamates, will predominantly arise through plastic rather than evolutionary processes. While plastic shifts towards earlier breeding dates may transiently enhance fitness, their long‐term sustainability will ultimately depend on the costs of reproducing under increasingly warmer thermal regimes. By providing the first quantitative estimate of the heritability of breeding dates in a wild reptile, our study highlights the urgent need to integrate both plastic and genetic components of breeding phenology into predictive frameworks of reptiles' responses to climate change.

## AUTHOR CONTRIBUTIONS

Théo Bodineau, Pierre de Villemereuil, Jean‐François Le Galliard and Sandrine Meylan conceived the ideas and designed the methodology; Pierre de Villemereuil, Alexis Rutschmann, Murielle Richard, Jean Clobert, Manuel Massot and Sandrine Meylan collected the data; Murielle Richard performed the molecular and statistical analyses necessary to build up the pedigree, with help from Alexis Rutschmann; Théo Bodineau and Pierre de Villemereuil analysed the data with the help of Jean‐François Le Galliard and Sandrine Meylan; Théo Bodineau led the writing of the manuscript with the help of Pierre de Villemereuil, Jean‐François Le Galliard and Sandrine Meylan. All authors contributed critically to the paper and gave final approval for publication.

## CONFLICT OF INTEREST STATEMENT

The authors declare no competing or financial interests.

## ETHICS STATEMENT

Experiments were performed under the approval of Cuvier Experimentation Ethics Committee (APAFIS#15897‐2018070615164391 v3 & APAFIS #34093‐2021112211094302 v4) in an animal breeding facility accredited by the Departmental Directorate for Employment, Labour, Solidarities and Population Protection with permit DDETSPP‐SPAE‐2022‐262‐001.

## STATEMENT ON INCLUSION

Our study brings together authors from the same countries, including senior researchers, a professor, a lecturer, postdoctoral researchers and an engineer. All authors were engaged early in the research and study design to ensure that the diverse sets of perspectives they represent were considered from the onset.

## Supporting information


**Table S1.** Output of statistical models used to quantify the shape, strength and temporal variation of natural selection on breeding phenology in the common lizard (see Table 2).
**Figure S1.** Temporal trend of (a) mean daily maximum temperature recorded from the 29th April to the 7th July (*T*_max_) and (b) mean parturition date (residuals of a linear regression between female parturition date and Rearing site) in our study population of common lizard (*Zootoca vivipara*) between 1989 and 2019.
**Figure S2.** Linear regressions between (a) minimum, (b) mean and (c) maximum daily temperatures recorded in our study site and the ones recorded at Mont Aigoual from August 2022 to 2023.

## Data Availability

Data are available at Zenodo: https://doi.org/10.5281/zenodo.17494327 (Bodineau et al., 2025).
