# Supplementary material for: High thermal plasticity, low heritability and constant directional selection on breeding phenology in a viviparous ectotherm
Source: J Anim Ecol. 2026 Aug 14;95(10):2133–46. doi: 10.1111/1365-2656.70335 (PMC13643182; doi:10.1111/1365-2656.70335)
Supplement: Supplementary file 1 [file JANE-95-2133-s001.docx]

***Journal of Animal Ecology***

**Supplementary information for**

High thermal plasticity, low heritability and constant directional selection on breeding phenology in a viviparous ectotherm

Table S1: Output of statistical models used to quantify the shape, strength and temporal variation of natural selection on breeding phenology in the common lizard (see Table 2).

| Hypothesis / Model | Parameter | Estimate | SE | z-value | p-value |
| --- | --- | --- | --- | --- | --- |
| No selection  Annual number of viable juveniles ~ SVL + Rearing site + (1\|Year) | (Intercept) | 1.23 | 0.09 | 13.74 | <0.0001 |
|  | SVL | 0.26 | 0.01 | 20.76 | <0.0001 |
|  | Rearing site - Pont de Montvert | 0.3 | 0.12 | 2.52 | 0.012 |
|  | Rearing site - Villefort 1 | 0.26 | 0.12 | 2.06 | 0.04 |
|  | Rearing site - Villefort 2 | 0.26 | 0.1 | 2.62 | 0.009 |
| Constant directional selection  Annual number of viable juveniles ~ SVL + Standardised parturition date + Rearing site + (1\|Year) | (Intercept) | 1.22 | 0.09 | 14.21 | <0.0001 |
|  | SVL | 0.24 | 0.01 | 18.86 | <0.0001 |
|  | Standardised parturition date | -0.05 | 0.02 | -2.81 | 0.00492 |
|  | Rearing site - Pont de Montvert | 0.3 | 0.11 | 2.65 | 0.00797 |
|  | Rearing site - Villefort 1 | 0.26 | 0.12 | 2.2 | 0.0275 |
|  | Rearing site - Villefort 2 | 0.26 | 0.1 | 2.74 | 0.006 |
| Constant quadratic selection  Annual number of viable juveniles ~ SVL + Standardised parturition date + Standardised parturition date² + Rearing site + (1\|Year) | (Intercept) | 1.23 | 0.09 | 14.08 | <0.0001 |
|  | SVL | 0.24 | 0.01 | 18.85 | <0.0001 |
|  | Standardised parturition date | -0.05 | 0.02 | -2.73 | 0.006 |
|  | Standardised parturition date² | 0 | 0.01 | -0.42 | 0.671 |
|  | Rearing site - Pont de Montvert | 0.3 | 0.11 | 2.62 | 0.009 |
|  | Rearing site - Villefort 1 | 0.26 | 0.12 | 2.19 | 0.029 |
|  | Rearing site - Villefort 2 | 0.26 | 0.1 | 2.72 | 0.007 |
| Fluctuating directional selection  Annual number of viable juveniles ~ SVL + Standardised parturition date + Rearing site + (Standardised parturition date\|Year)  model.zi.RS | (Intercept) | 1.24 | 0.08 | 15.51 | <0.0001 |
|  | SVL | 0.24 | 0.01 | 18.85 | <0.0001 |
|  | Standardised parturition date | -0.05 | 0.02 | -2.96 | 0.003 |
|  | Rearing site - Pont de Montvert | 0.29 | 0.11 | 2.72 | 0.007 |
|  | Rearing site - Villefort 1 | 0.2 | 0.11 | 1.78 | 0.075 |
|  | Rearing site - Villefort 2 | 0.26 | 0.09 | 2.94 | 0.003 |
| Constant selection mediated by Tmax  Annual number of viable juveniles ~ SVL + T_max_ + Rearing site + (1\|Year) | (Intercept) | 0.99 | 0.38 | 2.6 | 0.009 |
|  | SVL | 0.26 | 0.01 | 20.76 | <0.0001 |
|  | T_max_ | 0.02 | 0.03 | 0.63 | 0.53 |
|  | Rearing site - Pont de Montvert | 0.27 | 0.13 | 2.16 | 0.031 |
|  | Rearing site - Villefort 1 | 0.24 | 0.12 | 1.96 | 0.05 |
|  | Rearing site - Villefort 2 | 0.22 | 0.12 | 1.84 | 0.065 |
| Constant selection mediated by parturition date and Tmax  Annual number of viable juveniles ~ SVL + Standardised parturition date + T_max_ + Rearing site + (1\|Year) | (Intercept) | 1.35 | 0.39 | 3.47 | <0.001 |
|  | SVL | 0.24 | 0.01 | 18.7 | <0.0001 |
|  | Standardised parturition date | -0.05 | 0.02 | -2.75 | 0.006 |
|  | T_max_ | -0.01 | 0.03 | -0.33 | 0.745 |
|  | Rearing site - Pont de Montvert | 0.32 | 0.12 | 2.6 | 0.009 |
|  | Rearing site - Villefort 1 | 0.27 | 0.12 | 2.24 | 0.025 |
|  | Rearing site - Villefort 2 | 0.28 | 0.12 | 2.44 | 0.015 |

Note: Random effects are indicated in brackets. SVL: snout-vent-length, T_max_ : Mean daily maximum temperature between 29^th^ April and 7^th^ July

Figure S1: Temporal trend of (a) mean daily maximum temperature recorded from the 29^th^ April to the 7^th^ July (T_max_) and (b) mean parturition date (residuals of a linear regression between female parturition date and Rearing site) in our study population of common lizard (*Zootoca vivipara)* between 1989-2019. From 1989 to 2019, T_max_ increased by around 2.5°C in our study site (b = 0.08 ± 0.03 SE; F_1,29_ = 5.46, p = 0.005) but mean parturition date did not change significantly (linear change: F_1,26_ = 0.03, p = 0.87; quadratic change: F_1,25_ = 0.26, p = 0.61). Colours represent changes in Rearing sites during population monitoring. Points represent average daily maximum temperatures and parturition dates (± SD). The black linear regression line is plotted with its 95% confidence limits (shaded areas).

Figure S2: Linear regressions between (a) minimum, (b) mean and (c) maximum daily temperatures recorded in our study site and the ones recorded at Mont Aigoual from August 2022 to August 2023. The black regression line is plotted with their 95% confidence limits (shaded areas).
